# Potential pitfalls in the accuracy of analysis of natural sense-antisense RNA pairs by reverse transcription-PCR

**DOI:** 10.1186/1472-6750-7-21

**Published:** 2007-05-04

**Authors:** Fadia Haddad, Anqi X Qin, Julie M Giger, Hongyan Guo, Kenneth M Baldwin

**Affiliations:** 1 Physiology and Biophysics Department; University of California Irvine, Irvine, CA 92697; USA

## Abstract

**Background:**

The ability to accurately measure patterns of gene expression is essential in studying gene function. The reverse transcription polymerase chain reaction (RT-PCR) has become the method of choice for the detection and measurement of RNA expression patterns in both cells and small quantities of tissue. Our previous results show that there is a significant production of primer-independent cDNA synthesis using a popular RNase H^- ^RT enzyme. A PCR product was amplified from RT reactions that were carried out without addition of RT-primer. This finding jeopardizes the accuracy of RT-PCR when analyzing RNA that is expressed in both orientations. Current literature findings suggest that naturally occurring antisense expression is widespread in the mammalian transcriptome and consists of both coding and non-coding regulatory RNA. The primary purpose of this present study was to investigate the occurrence of primer-independent cDNA synthesis and how it may influence the accuracy of detection of sense-antisense RNA pairs.

**Results:**

Our findings on cellular RNA and *in vitro *synthesized RNA suggest that these products are likely the results of RNA self-priming to generate random cDNA products, which contributes to the loss of strand specificity. The use of RNase H^+ ^RT enzyme and carrying the RT reaction at high temperature (50°C) greatly improved the strand specificity of the RT-PCR detection.

**Conclusion:**

While RT PCR is a basic method used for the detection and quantification of RNA expression in cells, primer-independent cDNA synthesis can interfere with RT specificity, and may lead to misinterpretation of the results, especially when both sense and antisense RNA are expressed. For accurate interpretation of the results, it is essential to carry out the appropriate negative controls.

## Background

The ability to accurately measure patterns of gene expression is essential in studying gene function. RNA expression patterns can be determined using northern blots or RNase protection assays. However, these methods lack sensitivity and require large amounts of RNA sample. Therefore, they are not suitable in most cases especially when analyzing low expression levels of RNA. The reverse transcription-polymerase chain reaction (RT-PCR) has become the method of choice for the detection and measurement of RNA expression patterns in both cells and small quantities of tissue. The first step in this process consists of the cDNA synthesis in which a DNA complementary to the RNA is synthesized with the help of a reverse transcriptase enzyme in the presence of dNTP and DNA primers. An oligo dT primer is used to make cDNA of poly A-RNA such as mRNA. In contrast, random primers are used for making cDNA of all the RNA population including poly A-RNAs and ribosomal RNAs. In addition, gene specific primers that are complementary to a specific sequence of the RNA are often used for gene-specific RT. This latter method is commonly used for studying expression of RNAs that are thought to be expressed in both orientations. Recently, we found that cDNA synthesis can occur in the absence of primers in the RT-reaction [1]. The occurrence of cDNA synthesis in absence of RT primers or in the presence of false primers was documented previously [2], and it was attributed to a lack of stringency causing false priming or priming by other RNA molecules present in solution along with the target RNA [2]. This observation is problematic when detection of a specific RNA strand is needed. This strand-specificity problem was previously observed in the field of virology, whereby RT-PCR detection was associated with a high rate of false-positive results [3-6]. For example in viral RNA detection, the "+strand" is the viral genome, while the "- strand" RNA is indicative of active viral replication; therefore, stringent assays were designed to specifically detect one strand versus the other [3-6].

Recent studies show that bidirectional transcription is more widespread in the mammalian transcriptome than initially thought [7-17], which results in the expression of sense and antisense RNA pairs, which are either partially or completely overlapping. Therefore, the traditional assays may not be well suited for analyses of a specific strand of RNA. Antisense RNAs consist of either coding or non-coding RNAs, and are of diverse function affecting the corresponding sense gene regulation [14,18]. Understanding the integration of gene transcription and related complex regulatory mechanisms requires a better understanding of antisense RNA regulation. While RT PCR is a basic method used for the detection and quantification of RNA expression in cells, primer-independent cDNA synthesis can interfere with RT specificity, and may lead to misinterpretation of the results. Although this problem is known based on previous work in virology, it is often ignored in cell biology research dealing with gene expression. Instead, the concern is mostly focused on discrimination against genomic DNA contamination and/or carry-over DNA, which can give a false-positive PCR product [19-21].

Myosin heavy chain (MHC) is a key striated muscle motor protein which plays a major role in the regulation of the contraction process. MHC is expressed in several isoforms which are the products of distinct genes. These isoforms differ in their biochemical and functional properties. Two isoforms are expressed in heart muscle, namely α (MYH6) and β (MYH7). MYH6 and MYH7 genes are located in tandem on the same chromosome (#15 in rats, 14 in human and mice), and each gene is 25 kb separated by 4.5 kb of intergenic region. These genes' expression in cardiac myocytes is highly coordinated in an antithetical fashion, and a natural antisense RNA is involved in their coordinated regulation. In a recent study, we have encountered technical problems while analyzing sense and antisense RNA and pre-mRNA across the cardiac MYH genes [1]. The problem consisted of the formation of a PCR product from RNA samples that were reverse transcribed in the absence of any primers (see ref [1]). We designated this product as a background signal generated from nonspecifically reverse transcribed RNA. In order to generate quantitative analyses, one must account for the formation of this product; therefore, the signal was subtracted from the total signal generated with the specific RT primer followed by PCR. Results were expressed as a "net" value, which is the difference between the total and the "background" no primer product [1]. This background consisting of a no primer RT PCR signal, continues to be problematic when analyzing overlapping RNA which is a widespread phenomena in the mammalian transcriptome, and it applies to several gene clusters such as the cardiac MYH genes [1], the skeletal MYH genes cluster [22], the Hoxa genes [23], and thyroid receptor α (Thra) gene [24] among many others [9,14,17,18,25-28]. In order to obtain accurate detection and quantification of antisense RNA one needs to demonstrate strand specificity of the signal obtained by RT-PCR methodology. Therefore, it is important to investigate the origin of this aberrant "background" signal, and determine if conditions can be designed to eliminate its formation.

The primary purpose of this present study was to investigate the basis of amplification of PCR products following reverse transcription (RT) of the RNA in the absence of primers in the RT reaction. Our investigation using cellular RNA and *in vitro *synthesized RNA led us to believe that these products (primer-independent RT, but both RNA and reverse transcriptase dependent products) are likely the results of RNA secondary structure associated with self-priming to generate random cDNA products. The reverse transcriptase enzyme characteristics and reaction conditions affect primer-independent cDNA synthesis and this can be minimized or avoided with proper analytical conditions. These self-primed products may be a major source of the confounding error in the detection and quantification of RNA expression when the antisense RNA strand is also expressed.

## Results

### RNase H^- ^RT and primer-independent cDNA synthesis

When reverse transcription of total RNA used RNase H^- ^reverse transcriptase in the absence of RT-primers, PCR resulted in detection of a product only in reactions from tissue that expresses the target RNA [see Additional file 1]. PCR carried out on non-reverse transcribed RNAs gave no PCR product, confirming that the resulting RT products are legitimate products of cDNA and not the result of genomic DNA contamination of the samples. These results show that the primer-independent RT-PCR product is specific to the targeted sequence although the cDNA was synthesized without a primer.

If a gene locus is transcribed on only a single strand, RNA detection without primers will only add to the signal, but is not problematic since it retains its sequence specificity. In contrast, if a gene locus is transcribed on both strand, thereby producing sense and antisense RNA pairs, RT reactions using RNase H^- ^RT may not be stringent enough to demonstrate expression of a specific RNA strand if the complementary strand is also expressed. One way to insure strand specific expression is to use the net PCR signal between specifically primed cDNA and primer-independent cDNA synthesis under identical RT-PCR conditions.

### Reverse transcription and strand specific RNA detection: contribution of no-primer RT to specifically primed-RT PCR signal

**Figure 1:**
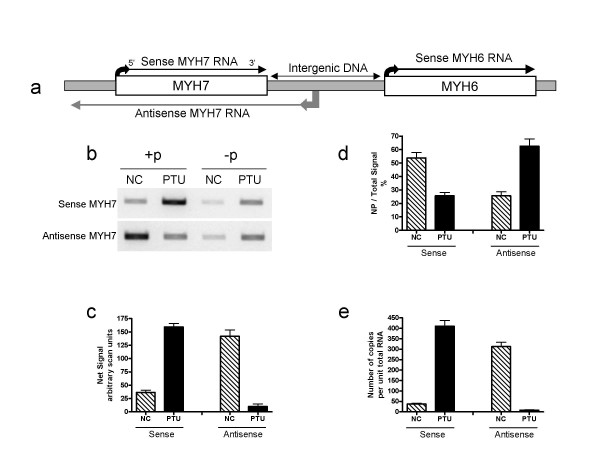
**RT-PCR in the analyses of naturally occurring endogenous sense-antisense RNA pair expression**. **(a) **Schematic of the cardiac MHC (MYH) gene locus and its transcription products. The upper strand transcribes the cardiac MYH7 and MYH6 sense RNA, the lower strand transcribes the antisense MYH7 RNA, which is abundant in normal control hearts [1]. **(b)**: representative gels obtained from RT-PCR targeting sense and antisense RNA corresponding to the MYH7 gene. RT used RNase H^- ^enzyme under manufacturer standard conditions (see methods) in presense of specific primers (+p) or in absence of primers (-p). **(c)** Bar graph depicting the net signal of MYH7 sense and antisense in each group, net consisting of the difference between +p and -p RT-PCR band intensity. Note that a normal control heart in the rat is associated with abundant relatively MYH7 gene expression (MYH6 gene expression is dominant). Under the PTU condition, MYH7sense RNA expression is increased. Antisense MYH7 RNA is strongly expressed in the normal control heart, based on strong net signal. In PTU heart, the antisense MYH7 RNA is decreased to a very low level. Note the +p product is similar to -p when targeting antisense MYH7 RNA in PTU hearts. **(d)** Bar graph depicting relative no-primer signal (NP) to the total signal in each group as determined by real time PCR methods. **(e)** Net MYH7 sense and antisense RNA copy numbers in NC and PTU hearts using real time PCR. Data are means ± SE. N = 6/group. See Additional file 4 for primer information. For both sense and antisense MYH7 targets, end-point PCR (b and c) used 0.2 μl of the cDNA and was performed for 28 cycles. For real time PCR, we used 320 nl cDNA for each sample, and the signal was compared to a standard curve established with a serial dilution of a standard consisting of purified PCR product as explained in the methods. See Additional file 4 for primer information. Based on standard curve linear regression analyses, copies for each target RNA were calculated.+p: a strand specific RT primer was included; -p: RT without primer. Sense is the amplification product of the sense target obtained when the reverse primer was added to the RT reaction. Antisense is the amplification product of the antisense target obtained when the forward primer was included in the RT reaction. In all these reactions, the presence of the no primer product depended on the presence of RNA and the RT enzyme, and was not formed in RT reactions that were carried out in the absence of the reverse transcriptase enzyme.

A strand-specific two-step RT-PCR approach was used to analyze the expression of the sense and antisense RNA products of the cardiac MYH7 gene (Fig 1) in rodent hearts undergoing cardiac MYH mRNA shifts from MYH6 to MYH7 expression such as in response to an induced hypothyroid state (i.e., by PTU treatment) [29,30]. RT reactions were carried out in parallel in either the absence of primers, or in the presence of gene-specific primers. These reverse transcriptions were carried out using RNase H^- ^reverse transcriptase (Superscript II) according to the recommended protocol (Invitrogen). Results show that the strand-specific RT-PCR reactions resulted in the formation of products. Sense MYH7 pre-mRNA was detected in the NC heart, and this was upregulated in the PTU-treated heart (Fig 1). In contrast, the antisense MYH7 RNA measured with the same PCR primer set showed a stronger signal for the normal control (NC) group than that of the PTU-treated group. RT reactions that were conducted without primers (-p) also generated measurable amounts of PCR products using the same PCR primers used to detect sense and antisense MYH7 RNA (Fig 1). These (-p) products lack strand specificity and may confound the results since they are upregulated with the PTU treatment. For example, although the antisense was detected in the PTU heart, a similar intensity product was formed from the non-primed reaction. When this product is accounted for by calculating the net expression, the PTU heart showed little antisense MYH7 RNA expression (Fig 1c). These observations make the results of RT-PCR questionable in the case when both sense and antisense RNA are expressed, i.e., requiring accuracy check by including the appropriate negative controls. Therefore, one needs to determine how much of the total signal is producded from specifically primed antisense RNA vs non-specifically primed (self primed) sense and antisense RNA.

These data in Fig 1b demonstrate that RNA strand specificity may be more difficult to establish when the target RNA is much less expressed than the complementary strand such as for the detection of the antisense MYH7 RNA in the PTU heart. Our results cannot determine for sure if the detection of the antisense MYH7 RNA is the result of noise due to the abundant presence of the complementary transcript or if it is the result of a true low expression as demonstrated by a low level of the net signal. Thus, knowing how much is the lower limit of detection of a specifically oriented RNA strand when the opposite strand RNA is abundantly co-expressed becomes an important issue to investigate.

To obtain a better quantitative measure on how much is the proportion of primer-independent signal to total signal, a real time PCR approach was utilized to analyze the MYH7 sense and antisense RNA products in NC and PTU treated hearts. Quantitative real time PCR was used to amplify cDNA generated in absence of primers and in presence of specific RT-primers targeting either the sense or the antisense RNA corresponding to the 5'end of the MYH7 gene in the rodent heart. Data analyses show that the contribution to the PCR signal of primer-independent cDNA relative to the specifically primed cDNA was different in the two experimental groups for the two target genes. For example, the proportion of no primer to total signal was high in the NC sample when targeting the sense RNA (MYH7 sense RNA), and this ratio decreased in the PTU heart (Fig 1d). In contrast, when targeting the antisense RNA species, the no-primer to total signal was low in NC, but increased in the PTU heart (Fig 1d). In these quantitative analyses, the number of copies of sense and antisense RNA was determined based on the net difference between signal generated from specific cDNA template and that generated from primer-independent cDNA template. The results for MYH7 RNA expression based on end point PCR as compared to real time PCR were similar (Fig 1c vs. 1e).

### *In vitro *RNA analyses by RT PCR: primer-independent versus primer-specific cDNA synthesis from *in vitro *overlapping RNA

**Figure 2:**
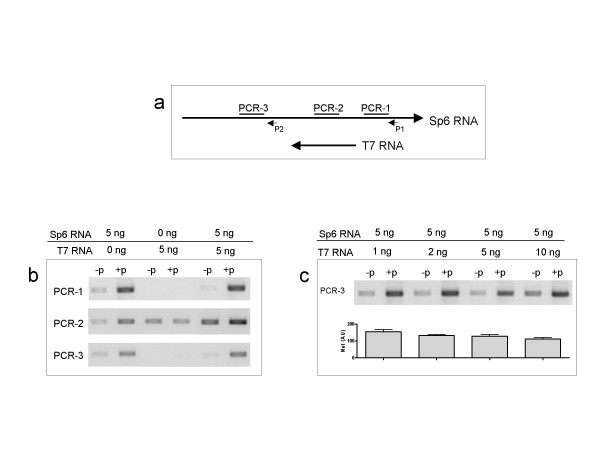
**RT PCR Analyses of pure in vitro sense and antisense RNA**. **(a) **Schematic diagram depicting the relative overlap between complementary *in vitro *RNA, Sp6 and T7 RNA. Position of the RT primer (P1 and P2) and of the PCR targets (PCR-1, PCR-2, and PCR-3) are also depicted. **(b)** Representative ethidium bromide stained gel results of RT-PCR. Sp6 and T7 RNA (5 ng) were reverse transcribed in 20 μl reaction either each one in pure form or mixed together. Reverse transcription was carried out in absence of any primers (-p), or in presence of Sp6-specific primer (+p); P1 for PCR1 and PCR-2; and P2 for PCR-3. PCR was performed on 100 nl cDNA for 22 cycles. **(c)** Sp6 RNA (5 ng) was reverse transcribed in 20 μl reaction mixed together with increasing amounts of T7 RNA as shown (from 1 to 10 ng). Reverse transcription was carried out in absence of any primers (-p), or in presence of Sp6-specific primer (+p, P1) followed by PCR using PCR-3 on 100 nl cDNA for 22 cycles. A representative ethidium bromide stained gel is shown, as well as a bar graph of the net signal [(+p)-(-p)] in arbitrary units (AU) as means+SE of 3 independent RT-PCR reactions. See Additional file 4 for primer information.

When we initially observed the formation of no-primer RT-PCR products during analyses of cardiac and skeletal muscle MYH genes' RNA, we thought that the primer-independent cDNA synthesis was caused by the presence of sense and antisense RNA pairs in solution. It was believed that the sense RNAs prime the antisense RNAs in the reverse transcription reaction, and vice versa. In order to test this hypothesis, we decided to synthesize sense and antisense RNA pairs *in vitro*, and examine their behaviors in RT-PCR in pure forms or in mixtures of known composition of sense and antisense RNA templates. A plasmid DNA vector was constructed so that after restricted digestions and *in vitro *RNA transcription one can obtain RNA strands that are complementary to each other as that encountered for overlapping genes [Additional file 2]. Two RNA strands were synthesized; Sp6 RNA that is 1207 bp, and T7 RNA that is 334 bp and is fully complementary to SP6 RNA with the overlap corresponding to sequences between 566 to 890 bp (Fig 2).

In order to examine the behavior of primer-independent cDNA synthesis in the absence and presence of the opposite strand RNA as well as in function of the PCR primers position, three solutions of *in vitro *RNA were tested: pure SP6, pure T7, and a mix of Sp6 and T7 in equal amounts. Reverse transcriptions were performed under two conditions: 1) in the absence of any primer (-p), and 2) in presence of a specific primer to Sp6 RNA (+p) (Fig 2). PCR reactions were performed with amplicon targets found on Sp6 alone such as PCR-1 and PCR-3, or on both Sp6 and T7 RNAs, such as PCR2 (Fig 2a). Our results show that a pure form of RNA is capable of primer-independent cDNA synthesis based on the formation of a PCR product in -p RT reactions (Fig 2b), and this product was obtained only when the RNA span the amplicon PCR target. For example, a no primer RT-PCR product was detected when pure Sp6 RNA was used as templates for all the tested PCR primer sets (Fig 2b). In contrast, when pure T7 RNA was used as template, only PCR primer set #2 yielded a PCR product (Fig 2b). In a mix of both Sp6 and T7 RNA, the -p RT-PCR signal for PCR2 was stronger than that obtained from each pure RNA tested alone (Fig 2b). An increase in the signal was evident in all specifically primed RT-PCR reactions when SP6 RNA was included either alone or in presence of T7 (Fig 2). To test the possibility that overlapping RNA may be in a position to prime the RT reaction to give a PCR product, we have tested different mixes of RNA in RT-PCR reactions whereby the PCR target sequence is in a position relevant to be a product of primed cDNA synthesis initiated by the overlapping RNA as depicted for primer set-3 in Fig 2. The data show that neither -p nor +p RT-PCR products were affected by the amount of overlapping RNA present in the reaction (Fig 2c). Therefore, the possibility that overlapping RNAs may be priming the RT reaction is not a valid explanation. Thus, it is more likely that the RNA is self primed to make self cDNA at random and perhaps driven by RNA folding and its secondary structure.

### RNase H activity of reverse transcriptase, reaction temperature, and RNA-strand specificity of RT-PCR

**Figure 3:**
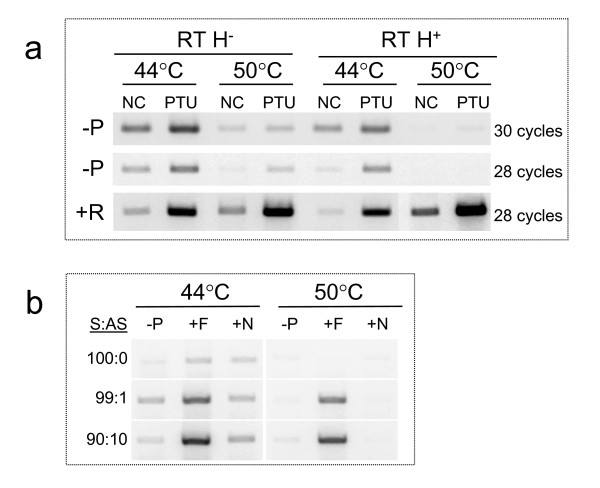
**Testing different RT enzyme properties and conditions**. **(a) **In a two-step RT-PCR system, 2 μg total RNA from NC and PTU treated hearts were reverse transcribed in 20 μl reactions in absence (-p) or presence (+p) of RT primers. The RT primer targeted the MYH7 sense RNA, and the PCR primer set amplified a 284 bp product corresponding to the 3' end of the MYH7 gene. PCR used 1 μl cDNA and was carried out for either 28 or 30 cycles. Shown are results from using two different RT enzymes that differed by their RNase H properties. RNase H^- ^and RNase H^+^. For each enzyme, the RT reactions were carried out under two different temperatures: 44°C or 50°C for 30 minutes/ea. **(b) **RT-PCR targeting the antisense MYH7 RNA in total RNA mixes of known proportions of sense and antisense RNA. RNA template contained either only sense MYH7 RNA, or a mix of sense and antisense MYH7 RNA corresponding to 99 to1 or 90 to 10 sense to antisense ratios (S:AS). Soleus total RNA was used as a source of the sense MYH7 RNA in absence of antisense. Whereas, T3-treated heart total RNA was used as a source of the antisense MYH7 RNA without co-expression of the sense. Mixes of soleus and T3 treated heart RNA were used to achieve the noted S:AS amounts in 2 μg of total RNA per 20 μl reactions. Reverse transcriptions were carried out in absence of RT primers (-P), in presence of the forward primer (+F) targeting the antisense, and in presence of a non specific primer corresponding to the 3' untranslated region of the human MYH4 mRNA sequence (+N). RT reactions used RNase H^- ^RT (Invitrogen), performed at 44°C or at 50°C for 30 min. PCR was carried out on 1 μcDNA for 28 cycles targeting the 3' end of the MYH7 gene. See Additional file 4 for primers information.

All the experiments reported above were performed with a popular reverse transcriptase, Superscript II, which is RNase H negative (^-^) at the recommended temperature by the manufacturer, 44°C. To answer the question on how the RNase H activity of the reverse transcriptase or the reaction temperature may affect the RNA strand-specificity of cDNA synthesis, we compared RNase H positive (^+^) RT (Omniscript, Qiagen) vs. RNase H^- ^RT (Superscript II, Invitrogen) specificity at two different temperatures, 44°C and 50°C, basing the comparison on RT-PCR results when the RT is carried out in the absence of primers. Under both temperature conditions, RNase H^- ^RT was associated with formation of more no primer product than the RNase H^+ ^RT (Fig 3). For each RT enzyme, specificity is improved when the RT reactions were carried out at a higher temperature (50°C vs 44°C), as judged by a reduction in the formation of the no primer RT-PCR product (Fig 3). This reduction in signal for the -P RT PCR was not the result of a reduced sensitivity as one might suggest. In fact, when parallel reactions were conducted in the presence of specific RT primer (+R), the obtained RT-PCR product was not reduced, and in some cases it was even increased (Fig 3a). In order to further investigate reaction specificity, RT PCR conditions were aimed to detect antisense MYH7 RNA in total cellular RNA preparations in presence of 10 or 100 fold complementary sense MYH7 RNA. RT reactions were carried out at both 44°C and 50°C under three RT-priming conditions: 1) in absence of any primers in the RT, 2) in presence of specific primer (F) complementary to antisense MYH7 RNA, and 3) in presence of a non specific primer (N) corresponding to human MYH4 gene and with little sequence similarity to the MYH7 gene. Results reported in Fig 3b showed that PCR products were formed in all RT-PCR reactions when the RTs were performed at 44°C. When no target RNA (MYH antisense) was in the template, there was no net product formation between the non specific RT vs. the specific RT-PCR. Although "background" RT-PCR products were formed in -P and +N RT, there was an increase in the RT-PCR signal when a specific RT primer was included. This increase was visible even when the target was only 1% relative to MYH7 template; the other 99% were complementary to the target RNA being analyzed. An improvement in specificity was established when the RT reactions were carried out at 50°C as demonstrated by a significant reduction in the background RT-PCR product formation (Fig 3b).

### Assessments of the One Step RT-PCR systems in strand-specific RNA analyses

**Figure 4:**
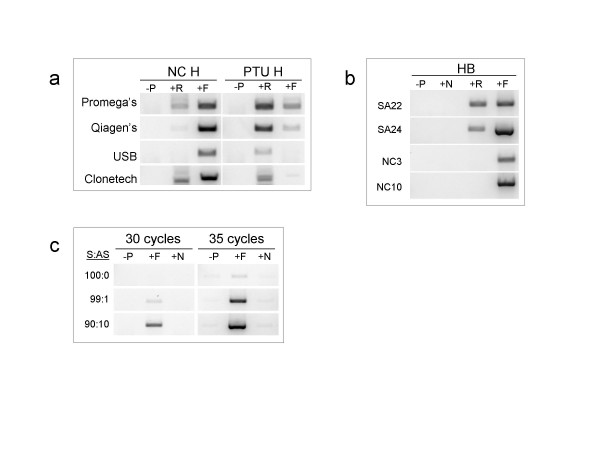
**One Step RT-PCR systems and specificity for the analyses of sense-antisense RNA pairs**. **(a) **Comparisons of four commercially available one step RT-PCR kits regarding their performance in analyses of sense and antisense MYH7 RNA expression in NC and PTU heart total RNA. -P is when the RT step was carried out in absence of primers. +R is when the RT was carried out in presence of reverse primer that is targeting the sense RNA. +F is when the RT was carried out in presence of forward primer that is targeting the antisense RNA. **(b)**. One Step RT-PCR using the Qiagen kit. Reactions used human brain RNA and PCR targeting either sense or antisense RNA of specific genes shown to be expressed in sense/antisense pairs (SA22, SA24) or in a single form (NC3, NC10). These primers were used previously by Chen et al., [9]. As a further test of specificity, the RT reactions were carried out in absence of primers (-P) or in presence of a non specific primer (+N), complementary to human MYH4 mRNA, MYH 4 is not expressed in the brain and is not related to any of the studied genes. **(c)** One step RT-PCR (Qiagen kit) used to detect small amount of antisense (AS) MYH 7 RNA in mixed total RNA preparations containing either 100% sense (S) MYH7 RNA, (100:0), or 99:1 and 90:10 of S:AS ratios. Sense MYH7 is derived from soleus muscle total RNA, whereas antisense MYH7 is derived from total RNA in T3-treated rat hearts. Reactions contained 100 ng of total RNA of the specified S:AS composition. RT reaction included either no primers (-P), a forward primer (+F) to target the antisense, and a non-specific primer (+N). For all these reactions in a, b, and c, 100 ng total RNA was used per reaction, and the RT was carried out at 50°C for 30 minutes followed by 10 minutes at 95°C. The PCR was carried out for 30 cycles (a, b, and c) or 35 cycles (c). See Additional file 4 for primer information.

In addition to the above two step-RT PCR systems we used, we also tested the one step-RT PCR system. Four different commercial kits were tested in their specificity and detection of sense and antisense MYH7 RNA in the rodent heart under normal and PTU treatment conditions (Fig 4a). The results of these experiments show that for all tested kits, the formation of a product depended on the inclusion of a specific primer in the RT reaction step. All four kits exhibited no product formation for the no primer RT (Fig 4a). While all these kits performed equally well in having little primer-independent cDNA synthesis, the pattern of expression of sense and antisense MYH7 RNA, as detected in these One step RT-PCR kits, was consistent with what is known about the regulation of this gene's RNA in response to altered thyroid state [31]. The Qiagen kit appeared to yield the sharpest and strongest product among all four tested kits (Fig 4) under the followed conditions. Furthermore, this kit (Qiagen One Step RT-PCR) appeared to perform well in strand specific RNA detection in human brain RNA when targeting certain genes that are expressed as sense and antisense pairs, or as a single orientation [9]. In addition to no-primer RT, a non-specific RT primer was added to the RT step, and it was found not to amplify any signal. Finally, the one step RT-PCR kit was tested in terms of its ability to specifically detect small amount of antisense RNA in presence of large amount of complementary sense RNA, in total cellular RNA preparations. Results shown in Fig 4c demonstrate that in a total RNA solution, RT-PCR can detect a specific RNA strand in presence of 100 times excess of the complementary RNA strand.

## Discussion

Our results show primer-independent cDNA synthesis may significantly contribute to the PCR signal when analyzing RNA expression. Primer-independent cDNA synthesis is comparable in nature to random primed cDNA synthesis, which does not distinguish between sense and antisense transcripts when RNA in both orientations is expressed. Practically, strand specificity of RT-PCR is established via the use of a specific primer in the reverse transcription reaction. This primer sequence is reverse complementary to the RNA strand of interest. Theoretically, the primer anneals to its target and the reverse transcriptase uses the RNA as a template to synthesize a complementary DNA (cDNA) extension product of the primer. The PCR reaction amplifies the cDNA to produce a DNA fragment, the intensity of which correlates with the initial amount of specific target RNA in the analyzed sample. Lack of strand specificity does not appear to be a concern when a gene locus is transcribed on only one strand, because the no primer RT-PCR product is sequence specific [Additional file 1]. However, when a gene locus is actively transcribed on both strands so that the RNA products may be forming sense and antisense RNA pairs, strand specificity becomes important if one were to study the expression and the regulation of each RNA product. Our findings show that using standard two-step RT PCR conditions may not generate accurate data in terms of strand specificity, especially when using RNase H^- ^reverse transcriptase. An essential check for strand specificity of the RT-PCR is to perform the RT on the same sample without inclusion of any primers (no primer RT), or with inclusion of non-specific primers that are not related to the target gene's sequence. If these RT reactions (no primer and non-specific primer RT) result in amplifiable PCR-products (Fig 3b), these products must be the result of non-specific cDNA synthesis, i.e., a cDNA that was somehow non-specifically generated from the RNA solution in presence of the reverse transcriptase enzyme. RNA is a dynamic polymer, which in solution folds to form loops and hairpins internally within itself. The conformation of the RNA may be associated with short stretches of double stranded RNA as a result of its secondary structure. These internal double stranded RNA segments may be recognized and extended by the RT enzyme to form cDNA products. This self-generated cDNA becomes amplified later in the PCR reaction and this process interferes with the accuracy of strand specificity when both strands are co-expressed.

We have shown that the use of a pure form of RNA that was *in vitro *synthesized and DNase treated, was also associated with this "background" no primer RT-PCR product (Fig 2). These findings confirmed the self-priming hypothesis as a cause of the no-primer RT-PCR product formation. One may raise the possibility that perhaps short DNA fragments resulting from the DNase treatment may have acted as primers for cDNA synthesis. This possibility is unlikely because after DNase treatment, the RNA solution was re-extracted with a phenol based reagent (Tri-Reagent, Molecular Research Center) and isopropanol precipitated under conditions that purify only RNA. All experiments used for this manuscript used RNA that was re-extracted after DNase treatment. However, for some additional samples, the DNase-treated RNA was further purified using a RNA clean-up protocol along with Qiagen RNA columns that exclude short nucleic acids (DNA and RNA < 200 nt) from the final elution. Primer-independent cDNA synthesis also occurred in these latter RNA preparations (data not shown), which eliminates the above possibility implicating DNA fragments for the no-primer RT-PCR product formation.

In most RT reactions, it is recommended to initially heat the RNA at 60 to 70°C to relax the RNA, but later the RNA is put on ice, and then after the reaction assembly, the reaction is carried out at 44°C. In our experience, the initial heating did not help reducing the self primed cDNA synthesis in the absence of primers. Instead, our data show that the temperature of the RT-reaction (50°C vs. 44°C) is more important in establishing specificity than the initial heat denaturing of the RNA. The limiting factor under these conditions would be the thermostability of the RT enzyme. In this context, it is worth mentioning that self priming of the RNA was recognized previously as a potential source of interference with reverse transcription reaction efficiency, and carrying the RT reaction at higher temperature was recommended as a way to improve the reaction efficiency [32,33].

In addition to the temperature, the type of the RT enzyme may either improve or worsen the strand specificity of the RT-PCR. Using RNase H^- ^RT was associated with more self primed cDNA products than when using RNase H^+ ^(Fig 3a). Our explanation for this is that the intrinsic RNase H activity gradually degrades the RNA as it is made into cDNA. Thus less RNA becomes available for self-priming as the reaction progresses. In contrast, in RNase H^- ^RT reactions, the RNA will stay longer in solution and this allows higher yields and significantly more full-length cDNA. However, this is associated with a greater chance for self priming of the long lived RNA, and thus creating a higher proportion on no-primer RT-PCR product as shown in Fig 3a.

When a gene is active on only a single strand, the corresponding RNA is expressed in a single orientation. In RT-PCR reactions targeting this gene, the product resulting from self primed RNA RT-PCR is much lower than the specifically primed RT-PCR product, and the resulting PCR product is sequence specific. Therefore, if one were to examine RNA expression in a unidirectional sense, without having to worry about the antisense RNA, the presence of an aberrant no-primer RT-PCR product is of little significance and will not affect interpretation of the results. However, when one is dealing with two complementary RNA strands, a sense and antisense strand that are not equally expressed, the non specific product may be formed in significant amounts to interfere with the detection and quantification of the minor RNA product (see Fig 1 and 3).

While RT PCR is a basic method used for the detection and quantification of RNA expression in cells, its results can be misleading due to this problem of primer-independent cDNA synthesis. Nowadays, with more and more antisense RNA in the genome being discovered, it is evident that analyses of strand specific RNA is something to worry about more often than one would think, and this applies to every field in biology. Carrying out the appropriate negative controls, enable one to insure specificity for the target RNA. Alternatively, the PCR primers can be designed to amplify target cDNA sequences that correspond specifically to a single strand RNA of the pair; i.e., not part of the overlapping sense and antisense RNA strands. However, this strategy may not work if the extent of RNA strands is still unknown, or if one of the RNA strands is fully overlapped by the other.

Of the several RT reaction strategies that we tested in this study, we found that the one step RT-PCR system appeared to be the most strand specific as it did not give any PCR product when no primers or a non-specific primer were included in the RT reaction. Shorter RT reaction duration (30 minutes) and higher temperature were likely contributing to strand specificity of the RT in this one step-system. Other factors such as the reaction composition may have contributed to this increased specificity. Importantly, we tested four different commercially available kits (Promega, Qiagen, USB, and Clontech), and all were found to be RNA strand specific under the conditions utilized (Fig 4a).

In the literature, with the new modern advances in computational biology, several reports have emerged on the abundance of overlapping genes and the natural occurrence of antisense RNA [7,9,17]. In several reports, the existence of overlapping genes and expression of complementary RNA products in cells and tissue were verified with strand specific RT-PCR. In some reports, these assays were performed with one step or two step-RT PCR with the appropriate inclusion of no primer-RT reactions [7,9,17,27,34,35]. In other reports, investigators verified the *in silico *findings concerning overlapping transcripts via using a 2 Step RT PCR System with RNase H^- ^RT. These verification reactions were performed without the negative control for strand specificity [15,36,37]. Based on the findings reported here, sense and antisense RNA pairs could have been analyzed more accurately using a different RT than RNase H^- ^RT. Alternatively, their results could have been confirmed by performing an appropriate negative control such as RT in absence of RT primers, or in presence of a non specific primer, followed by PCR.

## Conclusion

While RT PCR is a basic method used for the detection and quantification of RNA expression in cells, primer-independent cDNA synthesis can interfere with RT specificity, and may lead to misinterpretation of the results, especially when analyzing bidirectional RNA. Primer-independent cDNA synthesis occurs in most reverse transcriptase reactions, and this may interfere with strand specific cDNA synthesis when both sense and antisense RNA are expressed. Primer strand-specificity of the RT-PCR may be important in the new era of biology when new evidence is emerging rapidly supporting the expression of both sense and antisense strands of the genomic DNA. For accurate interpretation of the results, it is essential to carry out the appropriate negative controls.

## Methods

### RNA template

As templates for RT-PCR experiments, we used mammalian total cellular RNA and *in vitro *synthesized RNA.

#### Mammalian RNA

Total RNA from rat tissue was utilized. Young adult female Sprague Dawley rats (200 ± 5 g bw; N = 18) were utilized for this study (Taconic Farms). One group of animals (N = 6) was treated daily with propylthiouracyl (PTU, 12 mg/kg bw, IP). Another group (N = 6) was treated with triiodothyronine (T3, 150 μg/kg bw, IP), in order to induce either a hypothyroid or a hyperthyroid state, respectively. These altered thyroid states are associated with a marked change in cardiac myosin heavy chain (MHC) gene expression [31]. Animals were euthanized after 7 days of treatment, 6 hours after the last treatment (PTU or T3 injection), with an overdose of pentobarbital (100 mg/kg). The heart (ventricles), liver, and skeletal muscles (medial gastrocnemius and soleus) were excised, immediately frozen on dry ice and stored at -80°C until subsequent RNA extraction. Cardiac tissue total RNA obtained from animals treated with T3 was used as total RNA expressing MYH7 antisense RNA without co-expression of the sense, whereas soleus total RNA was utilized as the source of sense MYH7 RNA with undetectable amounts of the antisense. In addition to rat tissue RNA, some experiments utilized human brain total RNA purchased from Clonetech (Mountain View, CA). This study followed the NIH Animal Care Guidelines and was approved by the University of California Irvine, Animal Care and Use Committee. 

Tissue RNA was extracted using the tri-reagent procedure according to the manufacturer protocol (Molecular Research Center). Both, human brain and rat tissue total RNAs were treated with RQ1 DNase I (Promega) using 1 unit/μg total RNA at 37 degree C for 30 minutes. RNA extraction and DNase treatment were as described previously [1,22,38]. RNA quality was examined by agarose gel electrophoresis using 0.5 μg of total RNA and ethidium bromide staining. Good quality, non-degraded RNA, is characterized with the existence of 28S and 18S ribosomal RNA bands visible on the gel. Only good quality RNA was utilized for subsequent analyses.

#### In vitro RNA synthesis

To prepare a plasmid construct to be used as template for RNA synthesis, a 757 bp PCR fragment amplified from the rat genome was directly cloned into a TA cloning vector pGEM T easy (Promega) [see Additional file 2]. Based on the construct's restriction map analysis, Sp6 transcription after NaeI digestion generates 1206 bp RNA. T7 transcription after StuI digestion generates RNA that is 334 bp in size. These T7 generated transcripts are entirely overlapping with SP6 NaeI RNA (see Fig 2 and Additional file 2), and they are complementary to nucleotides sequence between 565 and 899 on Sp6 RNA. *In vitro *RNA was transcribed from linearized plasmid DNA using the Mega Script RNA kit from Ambion with either SP6 or T7 RNA polymerase. At the completion of the *in vitro *transcription, the sample was treated with turbo DNase I (Ambion) which effectively removes traces of the original Plasmid DNA. After DNase treatment, the RNA was extracted using the tri-reagent LS (Molecular Research Center).

The purified RNA was tested first for any plasmid contamination by PCR targeting specific regions and using 100 ng of pure RNA template. For the positive control for these tests and for each primer set, 50 ng plasmid (pGEM T easy containing the insert) was used as template. It is important to note that if the *in vitro *synthesized RNA is to be used for RT-PCR, it must be free of any precursor plasmid which can function effectively as template for the PCR. For all the tested PCR primers, the RNA preparation PCR reactions gave no product whereas the plasmids gave a strong signal of the expected size. These results ensured that the DNase treatment was effective, thus validating the use of these *in vitro *RNAs as templates for subsequent RT-PCR testing.

### Reverse transcription (RT)

Each RNA sample was subjected to reverse transcription under three different conditions [see Additional file 3]: 1) a specific RT whereby the RNA was reverse transcribed in the presence of specific primers reverse complementary to the target RNA. 2) A negative control to check for genomic DNA contamination or for reagent purity. The RNA was subjected to reverse transcription whereby the RT enzyme was omitted from the reaction, while all other ingredients were kept intact. Detection of a PCR product from these negative control reactions may be attributed to either reagent contamination with PCR products or RNA contamination with genomic DNA. This latter component is important for an unspliced RNA target which amplifies the same fragment size as the genomic DNA. The absence of such a product ensures the lack of contaminants as well as the effectiveness of DNase I treatment of the RNA. 3) A negative control was used to check for reverse transcriptase specificity. The RNA was subjected to the RT reactions in the absence of any RT primers, but all other ingredients of the reaction were included. This type of negative control is necessary especially when targeting overlapping RNA to insure that the signal generated is specific to the targeted RNA strand and not to the reverse overlapping RNA. In some experiments, an additional negative control was also included. These RT reactions were carried out in presence of a non-specific primer of a sequence that is unrelated to the target RNA. When total RNA was utilized, 2 μg were included into a 20 μl reaction. When *in vitro *synthesized RNA was utilized, 5–10 ng of *in vitro *RNA were included per 20 μl reaction.

For these analyses, we have tested two popular reverse transcriptases that differ with their RNase H activity. Superscript II (SSII, RNase H^-^) from Invitrogen is a modified version of Moloney Murine Leukemia Virus (MMLV) in which RNase H activity is reduced. Omniscript (RNase H^+^) from Qiagen is a genetically engineered heterodimeric enzyme with an intrinsic RNase H activity. For these RT reaction procedures, the manufacturer recommendations were strictly followed. When a specific RT primer was included it was at 5 pmoles/20 μl reaction volume.

In these RT-PCR analyses, the reverse transcription reactions were carried out under standard condition (as recommended by the manufacturer) and compared to a modified condition whereby the temperature of the reaction was increased, while the reaction time was decreased [see Additional file 3]. When a primer was included in the RT reactions, it was at 5 pmoles/reaction in a total volume of 20 μl.

### Polymerase chain reactions (PCR)

A two-step RT-PCR system was utilized for most of these analyses. The cDNA products were amplified by either end point PCR (using a Stratagene Robocycler), or with real time quantitative PCR (QPCR, using Stratagene Mx3000p). For some RT-PCR analyses, a one step-RT-PCR system was utilized. PCR primer pairs were designed using Primer Select/DNA Star software. Sequence information on all RT and PCR primers used in this report is shown in Additional file 4. When applicable, PCR products were separated by electrophoresis on 2% agarose 1xTAE gels and stained with ethidium bromide. The ultraviolet light-induced fluorescence of stained DNA was captured by a digital camera, and the band intensities were quantified by densitometry with ImageQuant software (Molecular Dynamics) on digitized images and were reported as arbitrary scan units.

#### End point PCR

Reactions were 25 μl in volume and included variable amounts of cDNA depending on target abundance along with 15 pmoles of each of the reverse (Rev) and forward (Fwd) primers and used Biolase thermostable Taq DNA polymerase as recommended by the manufacturer (Bioline). PCR started with an initial denaturing step of 3 min at 96°C, followed by a variable number of cycles (20–35 cycles) consisting of 1 minute at 96°C, 45 seconds at the annealing temperature, (variable depending on the primers Tm), 45 seconds at 72°C. When the different RT reactions of the same RNA samples were being compared, equal amounts of cDNA were included in the PCR reaction under the same reaction and cycling conditions.

#### Quantitative real time PCR (QPCR) using SYBR green

Real time PCR was performed in order to obtain a quantitative relationship between PCR product obtained from primer-independent cDNA and that obtained with specifically primed cDNA. As template for these analyses, we used control and PTU heart cDNA and we targeted the cardiac MHC RNA expression, a condition associated with significant change in MHC gene expression [31]. Quantitative PCR was performed using the Mx3000p thermal cycler system and the FullVelocityTM SYBR^® ^Green QPCR master mix reagents from Stratagene (La Jolla, CA). A PCR primer set targeting the 5'end of the β MHC gene was used for these experiments [see Additional file 4 for primer information]. The primer set amplifies a 425 bp fragment, which was purified from the gel using a Qiagen gel extraction Kit. This DNA amplicon served as a standard for PCR amplifying cDNA from NC and PTU hearts. PCR specificity was judged based on the presence of a single product at the end of the 40 cycles based on melting curve analyses showing a single peak at the product melting temperature, as well as based on examination of the products after gel electrophoresis on 2% agarose gel and ethidium bromide staining. A serial dilution of the standard was established and used as template for QPCR. The primer concentration was optimized in order to achieve QPCR efficiency near 100%. For each sample, 320 nl of cDNA were amplified along with a serial dilution of the standard in order to verify the efficiency and linearity of the reaction. All standards and unknown samples were run in duplicates. As a negative control, some QPCR reactions were run in the absence of template cDNA, using water as template. For standard curve analyses, the Ct was plotted against the corresponding Log of input copies and the data was analyzed using linear regression. The equivalent cDNA copies for each sample were estimated based on linear regression analysis.

#### One step-RT-PCR

In addition to the above described two step RT-PCR reaction systems, a one step RT-PCR assay was also tested using four commercially available kits which were obtained from Qiagen, Promega, USB, and Clontech. In these reactions the RT and PCR were performed in a single tube in the following sequence: the RT reactions were performed at 50°C for 30 minutes under specified primer conditions, followed by 15 minutes heating at 95°C. At this stage, the missing PCR primers were added to the reaction, and PCR was conducted for a certain number of cycles depending on the target abundance. For each RNA sample four different reactions were carried out as follows which differed in their RT design: 1) no primers or non specific primers unrelated to the target RNA, were added to the RT reaction; 2) Either, the reverse PCR primer alone, or 3) the forward PCR primer alone were included in the RT reaction; 4) the RT enzyme was immediately denatured at 95°C for 15 minutes followed by adding the RNA template and PCR primers before performing the PCR. Under these last conditions, a product formation would be indicative of either genomic DNA contamination in the RNA sample or reagent contamination with the PCR product. For these One Step RT PCR reactions, each reaction was performed using 50–200 ng total RNA, and the PCR was carried out for 20–35 cycles depending on the abundance of the target RNA. Conditions to be compared were run on the same samples under similar conditions (template amounts, PCR cycle numbers).

## Authors' contributions

FH made the initial observation of primer-independent cDNA synthesis, and she contributed to the experimental design, analyses and interpretation of the data and drafted the manuscript. AXQ performed the plasmid construction for the *in vitro *RNA synthesis, and she participated in RT-PCR data generation and analyses. JG participated in the experimental design, data analyses, and interpretation of the results; as well as editing the manuscript. HG participated in the RNA extraction as well as real time PCR data generation and analyses. KMB contributed to the experimental design, interpretation of the results, and the writing of the manuscript. All authors read and approved the final manuscript

## Supplementary Material

Additional file 1 **Primer-independent cDNA synthesis and RT-PCR sequence specificity**. **(a) **2 μg total RNA were reverse transcribed in the absence of any primers in 20 μl reactions using RNase H^- ^reverse transcriptase (Superscript II) and total RNA from rat tissue including heart (H), liver (L), white medial gastrocnemius (WMG), and soleus (S) muscle. PCR was performed targeting the18S rRNA, the cardiac beta MHC RNA (MYH7), the cardiac alpha MHC RNA (MYH6), and the alpha skeletal actin RNA (ACTA). PCR was carried out for 30 cycles on various amounts of cDNA for each PCR primer set. (50 nl for 18S, 1 μl for MYH7 and ACTA, and 0.2 μl for the MYH6). Shown are ethidium bromide stained agarose gels depicting the product of such PCR reactions. Note that the selection of PCR primers was based on differential expression of their targets in the tested tissue RNA. For example, 18S rRNA is ubiquitously expressed, while the MYH7 RNA is expressed in the heart and soleus muscle but not in the liver and WMG muscle. The MYH6 RNA is expressed exclusively in the heart, while ACTA RNA is expressed only in WMG and soleus muscle. A small level of skeletal actin expression may also occur in the heart. **(b) **Human brain (HB) RNA analyses using primer-independent RT PCR. Representative images of ethidium bromide stained gels depicting the PCR products from primer-independent cDNA synthesis in a two-step RT-PCR system. In the RT, 2 μg total RNA was used in 20 μl reaction with RNase H- reverse transcriptase. PCR used 1 μl cDNA and was carried out for 30 cycles. Primers in **b **are based on Fig 2 and Supplementary Table 2 in Chen J. et al., (reference #9 in the main manuscript). Shown are replicates of the same RNA sample. SA: gene expressed as sense and antisense RNA pairs. NC: gene expressed as a single strand RNA, with no complementary RNA. See Additional file 4 for primer information. Click here for file

Additional file 2 **Schematic of the plasmid construct used for the *in vitro *RNA synthesis to generate complementary RNA. **A PCR product (757 bp DNA, hatched pattern), was ligated to pGEM-T easy between T7 and Sp6 promoters using T/A cloning technique (Promega). The construct was linearized by digestion with restriction enzymes which cut at specific sites, e.g., NaeI at position 3465, or StuI at position 334 This was followed by RNA synthesis using T7 or Sp6 RNA polymerase (Mega Script RNA kit from Ambion). +1 is the start of T7 RNA transcription. MCS: multicloning site region of pGEM-T Easy vector. Click here for file

Additional file 3 Description of the different RT reaction conditions that we used for the two step RT-PCR systems. Click here for file

Additional file 4 **RT and PCR primer information**. RT and PCR primer sequences, their specific target, PCR product size, and reference figure whereby these primers were used. Click here for file
