# Supplementary figures and images for: Potential pitfalls in the accuracy of analysis of natural sense-antisense RNA pairs by reverse transcription-PCR
Source: BMC Biotechnol. 2007 May 4;7:21. doi: 10.1186/1472-6750-7-21 (PMC1876213; doi:10.1186/1472-6750-7-21)

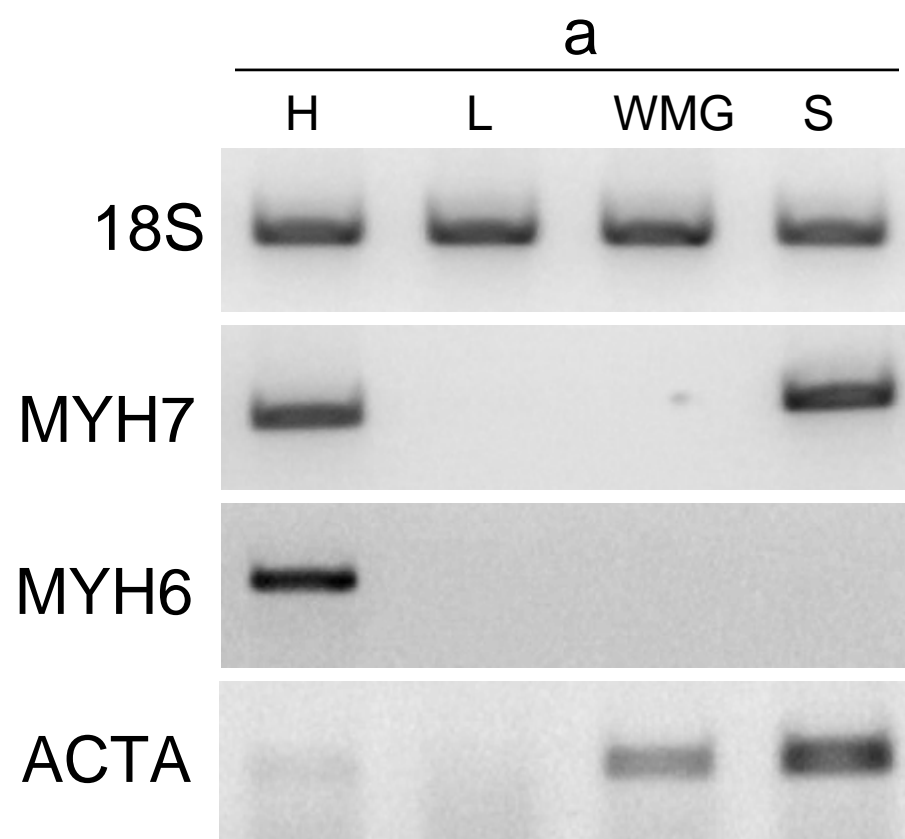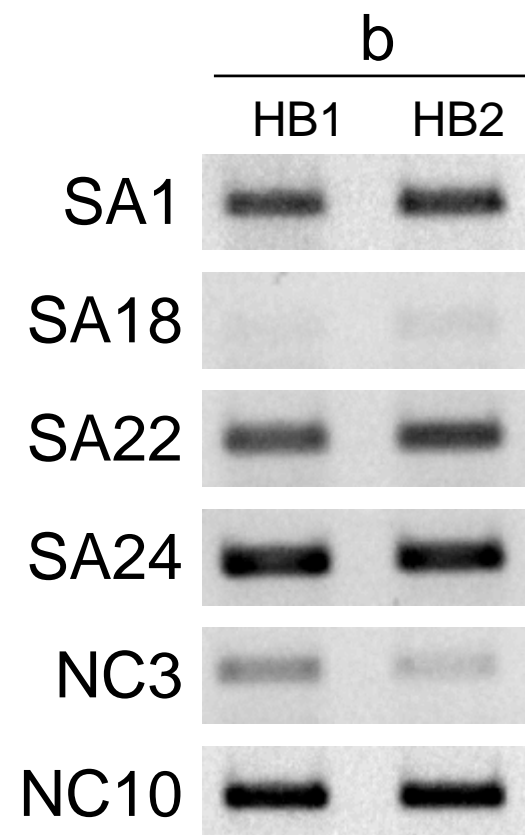

Supplement: Additional file 1 — Primer-independent cDNA synthesis and RT-PCR sequence specificity. (a) 2 μg total RNA were reverse transcribed in the absence of any primers in 20 μl reactions using RNase H- reverse transcriptase (Superscript II) and total RNA from rat tissue including heart (H), liver (L), white medial gastrocnemius (WMG), and soleus (S) muscle. PCR was performed targeting the18S rRNA, the cardiac beta MHC RNA (MYH7), the cardiac alpha MHC RNA (MYH6), and the alpha skeletal actin RNA (ACTA). PCR was carried out for 30 cycles on various amounts of cDNA for each PCR primer set. (50 nl for 18S, 1 μl for MYH7 and ACTA, and 0.2 μl for the MYH6). Shown are ethidium bromide stained agarose gels depicting the product of such PCR reactions. Note that the selection of PCR primers was based on differential expression of their targets in the tested tissue RNA. For example, 18S rRNA is ubiquitously expressed, while the MYH7 RNA is expressed in the heart and soleus muscle but not in the liver and WMG muscle. The MYH6 RNA is expressed exclusively in the heart, while ACTA RNA is expressed only in WMG and soleus muscle. A small level of skeletal actin expression may also occur in the heart. (b) Human brain (HB) RNA analyses using primer-independent RT PCR. Representative images of ethidium bromide stained gels depicting the PCR products from primer-independent cDNA synthesis in a two-step RT-PCR system. In the RT, 2 μg total RNA was used in 20 μl reaction with RNase H- reverse transcriptase. PCR used 1 μl cDNA and was carried out for 30 cycles. Primers in b are based on Fig 2 and Supplementary Table 2 in Chen J. et al., (reference #9 in the main manuscript). Shown are replicates of the same RNA sample. SA: gene expressed as sense and antisense RNA pairs. NC: gene expressed as a single strand RNA, with no complementary RNA. See Additional file 4 for primer information. [file 1472-6750-7-21-S1.pdf]

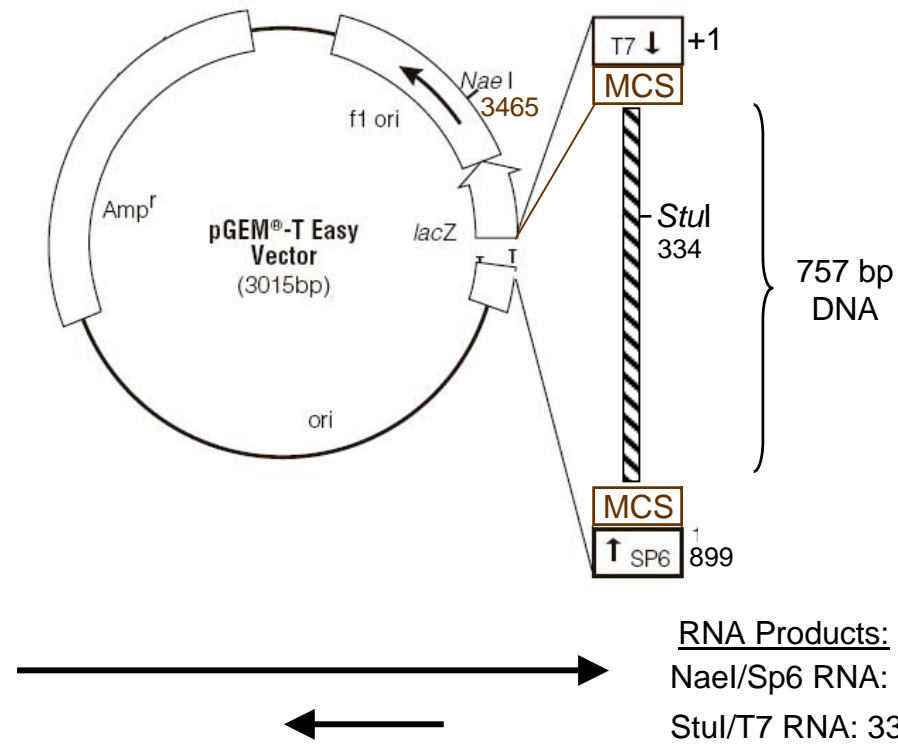

Supplement: Additional file 2 — Schematic of the plasmid construct used for the in vitro RNA synthesis to generate complementary RNA. A PCR product (757 bp DNA, hatched pattern), was ligated to pGEM-T easy between T7 and Sp6 promoters using T/A cloning technique (Promega). The construct was linearized by digestion with restriction enzymes which cut at specific sites, e.g., NaeI at position 3465, or StuI at position 334 This was followed by RNA synthesis using T7 or Sp6 RNA polymerase (Mega Script RNA kit from Ambion). +1 is the start of T7 RNA transcription. MCS: multicloning site region of pGEM-T Easy vector. [file 1472-6750-7-21-S2.pdf]
