# Supplementary material for: Potential pitfalls in the accuracy of analysis of natural sense-antisense RNA pairs by reverse transcription-PCR
Source: BMC Biotechnol. 2007 May 4;7:21. doi: 10.1186/1472-6750-7-21 (PMC1876213; doi:10.1186/1472-6750-7-21)
Supplement: Additional file 3 — Description of the different RT reaction conditions that we used for the two step RT-PCR systems. [file 1472-6750-7-21-S3.pdf]

	1) +RT primer, +enzyme		2) +RT primer, -enzyme		3) -RT primer, +enzyme	
RNase H^-<br><br>or<br><br>RNase H^+<br>RT enzyme	**A: Standard Condition.** Denature RNA+primer at 70°C, add RT reagents and preincubate at 44°C for 3’, add enzyme, incubate at 44°C for either 30 or 50 min	**B: Modified Condition.** Assemble reactions on ice, incubate at 50°C for 30 min	**A: Standard Condition.** Denature RNA+primer at 70°C, add RT reagents except the enzyme, incubate at 44°C for either 30 or 50 min	**B: Modified Condition.** Assemble reactions on ice by adding all reagents, RNA, and primers except the enzyme, incubate at 50°C for 30 min	**A: Standard Condition.** Denature RNA at 70°C, add RT reagents and except the primer, preincubate at 44°C for 3’, add enzyme, incubate at 44°C for either 30 or 50 min	**B: Modified Condition.** Assemble reactions on ice by adding all reagents, RNA, and enzyme except the, primers, incubate at 50°C for 30 min
