# Supplementary material for: Potential pitfalls in the accuracy of analysis of natural sense-antisense RNA pairs by reverse transcription-PCR
Source: BMC Biotechnol. 2007 May 4;7:21. doi: 10.1186/1472-6750-7-21 (PMC1876213; doi:10.1186/1472-6750-7-21)
Supplement: Additional file 4 — RT and PCR primer information. RT and PCR primer sequences, their specific target, PCR product size, and reference figure whereby these primers were used. [file 1472-6750-7-21-S4.pdf]

**Target**	**RT-PCR primers: 5'→3'**	**PCR product size (bp)**	**Reference figure**
18S rRNA	Fwd: AGGAATTGACGGAAGGGCAC<br>Rev: GTGCAGCCCCGGACATCTAAG	324	PCR in **Additional file 1**
5'end of the ACTA (αsk actin) gene	Fwd: GGCTGGGGGAGAGGAGTGT<br>Rev: CCCTGGGGGCATCATCC	210	PCR in **Additional file 1**
Antisense MYH7 RNA (5'end of gene)	TCACGTAGTACCAGGTTATTATTG		RT primer in Figures 1 and 3a at 44°C
Sense MYH7 RNA (5'end of gene)	CATGTACTGATAGGCGTTGTC		RT primer in Figures 1
5'end of MYH7 gene	Fwd: GCCTGGGCCTACCTCTTTATCC<br>Rev: GTACGCCGCCTTCCACCAC	425	PCR in Figures 1, **Additional file 1**, 3 and 4
3'end of MYH6 gene	Fwd: TCCTGCCCTCGAATTACCAGTCAT<br>Rev: ATAGCAACAGCGAGGCTCTTTCTG	399	PCR in **Additional file 1**
3'end of MYH7 gene	Fwd: AGCCAGGCCTGCTACCAAAGACACC<br>Rev: CCCCGGAACACCACGGAGCAT	284	PCR in Figures 3b, 4a, and 4c
Sense MYH4 RNA (3'end of gene)	CTTTGGTCACATTTTCTTTCATTAG		RT primer (N) in Figures 3 and 4
Sp6 RNA	GAGAAAGGAAGGGAAGAAAG		RT primer P1 in Figure 2
Sp6 RNA	TGTATAGGGCATATCTCTGGTG		RT primer P2 in Figure 2
PCR1 *in vitro* Sp6 RNA	Fwd: ATTACAATTCACTGGCCGTCGTTTTAC<br>Rev: CTAGGGCGCTGGCAAGTGTAGC	248	PCR1 in Figure 2
PCR2 *in vitro* Sp6 RNA	Fwd: GCCAGGCCTGCTACCAAAGACAC<br>Rev: GATCGGAACACCACGGAGCATAAAC	283	PCR2 in Figure2
PCR3 *in vitro* Sp6 RNA	Fwd: CTGGCCCAGAAGAGCATGACTGAGTT<br>Rev: GAGGGTACATGTCAAGGGGGAACACA	245	PCR3 in Figure 2
